# HS-1371, a novel kinase inhibitor of RIP3-mediated necroptosis

**DOI:** 10.1038/s12276-018-0152-8

**Published:** 2018-09-20

**Authors:** Han-Hee Park, Se-Yeon Park, Shinmee Mah, Jung-Hee Park, Soon-Sun Hong, Sungwoo Hong, You-Sun Kim

**Affiliations:** 1 Department of Biochemistry, Ajou University School of Medicine, Suwon, Korea; 2 Department of Biomedical Sciences, Graduate School, Suwon, Korea; 3 Center for Catalytic Hydrocarbon Functionalizations, Institute for Basic Science (IBS), Daejeon, 34141 Korea; 4 Department of Chemistry, Korea Advanced Institute of Science and Technology (KAIST), Daejeon, 34141 Korea; 5 Department of Biomedical Sciences, College of Medicine, Inha University, Incheon, 22332 Korea

## Abstract

Necroptosis is a type of programmed cell death that usually occurs under apoptosis-deficient conditions. Receptor-interacting protein kinase-3 (RIP3, or RIPK3) is a central player in necroptosis, and its kinase activity is essential for downstream necroptotic signaling events. Since RIP3 kinase activity has been associated with various diseases, the development of specific RIP3 inhibitors is an attractive strategy for therapeutic application. In this study, we identified a potent RIP3 inhibitor, HS-1371, by the extensive screening of chemical libraries focused on kinases. HS-1371 directly binds to RIP3 in an ATP-competitive and time-independent manner, providing a mechanism of action. Moreover, the compound inhibited TNF-induced necroptosis but did not inhibit TNF-induced apoptosis, indicating that this novel inhibitor has a specific inhibitory effect on RIP3-mediated necroptosis via the suppression of RIP3 kinase activity. Our results suggest that HS-1371 could serve as a potential preventive or therapeutic agent for diseases involving RIP3 hyperactivation.

## Cell death: Blocking the self-destruct mechanism

Researchers have identified a compound that inhibits necroptosis, a type of programmed cell death that occurs naturally but that can be harmful when overactivated. Necroptosis helps defend against disease, triggering virus-infected cells to self-destruct; however, hyperactivation of the mechanism is associated with inflammatory disorders such as inflammatory bowel disease. Triggering necroptosis requires a protein named RIP3, and Han-Hee Park at Ajou University, Suwon, South Korea and coworkers screened extensive chemical libraries to identify compounds that inhibit RIP3. They identified four compounds, and further testing showed that one, named HS-1371, strongly and specifically inhibited necroptosis in cells. HS-1371 could inhibit necroptosis even after the process had already begun, greatly broadening its therapeutic applications. HS-1371 may also help in treating other conditions that involve hyperactivation of necroptosis, including sepsis and multiple sclerosis.

## Introduction

Necroptosis has been well established as an important form of programmed cell death. It can be initiated by many cellular stressors, including signaling events activated by death receptor ligands, such as tumor-necrosis factor (TNF), TNF-related apoptosis-inducing ligand (TRAIL), or Fas ligand (FasL)^1–3^. Necroptosis is distinguished from apoptosis, which has been thought to occur without triggering inflammatory responses, in that it is highly pro-inflammatory. Necroptosis plays an important role in many pathological processes such as ischemia-reperfusion injury and host defense against viral infection^4–8^. Receptor-interacting protein kinase-3 (RIP3, or RIPK3) has been identified as a key player in necroptosis^9–11^, and the kinase activity of RIP3 is required for downstream signaling events including the recruitment of mixed lineage kinase domain-like protein (MLKL)^12–15^. Consistent with this finding, RIP3-kinase dead mutant D160N is unable to induce necroptosis^16,17^, indicating that RIP3 catalytic activity is indispensable for necroptotic cell death.

Our recent study showed that DNA-damaging agents activate RIP3-dependent necroptosis in cancer cells, and MLKL phosphorylation induced by DNA-damaging agents is dependent on RIP3 kinase activity^18,19^. Moreover, Geserick et al. proposed that strategies to upregulate RIP3 expression may activate the necroptotic signaling machinery in melanoma and that activation of the RIP3/MLKL pathway could be a treatment option for metastatic melanoma^20^. These studies suggest that the regulation of RIP3 kinase activity is important in cancer cell death. It has been reported that the compound, dabrafenib, interferes with MLKL phosphorylation and necroptosis through the suppression of RIP3 kinase activity as an off-target effect^21^, as dabrafenib is approved as a treatment for patients with B-RAF V600E mutation-positive advanced melanoma^22,23^. Inhibitors of V600E-mutated or V600K-mutated proto-oncogene serine/threonine protein kinase B-RAF (e.g., vemurafenib or dabrafenib) suppress the proliferation of BRAF-mutated melanoma cells^24^ and have significantly improved patient survival^25^. However, since RIP3 kinase activation potentiates melanoma cell death, the off-target effects of dabrafenib are potential issues for patients with B-RAF V600E mutation-positive advanced melanoma. We also reported that dabrafenib is a potential therapeutic agent for toxic epidermal necrolysis (TEN) via the inhibition of RIP3-mediated MLKL phosphorylation-induced necroptosis^26^. Although the regulation of RIP3 kinase activity has controversial effects on various diseases conditions^27^, novel RIP3 kinase inhibitors will undoubtedly be useful in the clinic.

In this study, we discovered potent RIP3 inhibitors by extensive cross-screening of our kinase-targeted chemical libraries and found that HS-1371 is a potent RIP3 kinase inhibitor. HS-1371 binds to the ATP binding pocket of RIP3 and inhibits ATP binding to prevent RIP3 enzymatic activity in vitro. Therefore, the inhibition of RIP3 kinase activity by HS-1371 protects cells from RIP3-mediated necroptosis. This novel RIP3 kinase inhibitor could be used as a therapeutic agent for diseases involving RIP3 hyperactivation.

## Materials and methods

### Preparation of HS-1371

7-(1-(Piperidin-4-yl)-1*H*-pyrazol-4-yl)-4-(*p*-tolyloxy)quinoline (HS-1371) was synthesized by Suzuki coupling followed by a Boc-deprotection step. 4-Phenoxyquinoline starting material and boronic ester reagent as a coupling partner for Suzuki coupling were prepared by S_N_Ar and miyaura borylation^28–30^.

1. Preparation of 4-phenoxyquinoline starting material. 4-Phenoxy-7-bromo-4-chloroquinoline (100 mg, 0.412 mmol), *p*-cresol (44.6 mg, 0.412 mmol), and K_2_CO_3_ (142 mg, 1.03 mmol) were dissolved in *N*,*N-*dimethylformamide (1.5 mL) under an N_2_ atmosphere. The reaction mixture was stirred for 12 h at 140 °C. After cooling to room temperature, the organic phase was diluted and extracted with EtOAc (100 mL × 3) from the aqueous layer. The combined organic phases were dried over anhydrous MgSO_4_ and filtered. The organic layer was purified using flash column chromatography (dichloromethane/methanol = 40:1) to give 7-bromo-4-(*p*-tolyloxy)quinoline (115 mg, 88%).

2. Preparation of boronic ester reagent. *Tert*-butyl 4-hydroxypiperidine-1-carboxylate (2.0 g, 9.94 mmol) solution in dichloromethane (30 mL) was added to triethylamine (1.4 mL, 9.94 mmol), and methane sulfonyl chloride (774 μL, 9.94 mmol) and 4-dimethylaminopyridine (122 mg, 3.98 mmol) were then added at 0 °C. The reaction mixture was stirred at room temperature for 14 h. Water was added to the reaction mixture at 0 °C, and the organic compounds were extracted with dichloromethane (100 mL × 3) followed by drying over Na_2_SO_4_. The combined organic layers were concentrated under reduced pressure, and the residue was purified using flash column chromatography (hexanes/EtOAc = 2:1) to afford *tert*-butyl 4-((methylsulfonyl)oxy)piperidine-1-carboxylate (2.71 g, 96%).

Sodium hydride (60% in mineral oil, 198 mg, 4.95 mmol) was added to 4-iodopyrazole (800 mg, 4.12 mmol) solution in *N*,*N-*dimethylformamide (16 mL) at 0 °C, and the reaction mixture was stirred for 1 h in a water bath. At room temperature, *tert*-butyl 4-((methylsulfonyl)oxy)piperidine-1-carboxylate (1.27 g, 4.54 mmol) was added, and the resulting mixture was stirred at 100 °C for 16 h. After quenching with water at 0 °C, organic compounds were extracted with EtOAc (200 mL × 3) and dried over anhydrous MgSO_4_ followed by concentration in vacuo. Purification using flash column chromatography (hexane/EtOAc = 2:1) afforded *tert*-butyl 4-(4-iodo-1*H*-pyrazol-1-yl)piperidine-1-carboxylate (1.21 g, 78%).

*Tert*-butyl 4-(4-iodo-1*H*-pyrazol-1-yl)piperidine-1-carboxylate (1.19 g, 3.16 mmol) was placed in a two-neck round-bottom flask equipped with a magnetic bar. A three-way stop cock was connected to one neck, and the other neck was sealed with an Aldrich septum. The starting material and flask were dried in vacuo and purged with nitrogen gas. Anhydrous tetrahydrofuran (12.6 mL) was added, and the solution was cooled to 0 °C. Isopropylmagnesium chloride solution (2 M) in tetrahydrofuran (2.4 mL) was added dropwise, and the mixture was stirred for 10 min at 0 °C. The mixture was warmed to room temperature and stirred for 1 h. Next, 2-methoxy-4,4,5,5-tetramethyl-1,3,2-dioxaborolane (0.803 mL, 4.90 mmol) was added at 0°C, and the resulting mixture was stirred at room temperature for 14 h. After the reaction was complete, a saturated aqueous NaCl solution was added at 0 °C, and organic materials were extracted with EtOAc (200 mL × 3), followed by drying over Na_2_SO_4_. Purification using flash column chromatography (hexane/EtOAc = 2:1) afforded *tert*-butyl 4-(4-(4,4,5,5-tetramethyl-1,3,2-dioxaborolan-2-yl)-1*H*-pyrazol-1-yl)piperidine-1-carboxylate (891 mg, 75%).

3. Suzuki coupling and deprotection of the Boc group. The mixture of 7-bromo-4-(*p*-tolyloxy)quinoline (23.9 mg, 0.0761 mmol), *tert*-butyl 4-(4-(4,4,5,5-tetramethyl-1,3,2-dioxaborolan-2-yl)-1*H*-pyrazol-1-yl)piperidine-1-carboxylate (34.4 mg, 0.0913 mmol), Pd(dppf)Cl_2_.CH_2_Cl_2_ (3.1 mg, 0.0038 mmol) and Cs_2_CO_3_ (81.8 mg, 0.251 mmol) in toluene:H_2_O ( = 2:1, toluene 0.5 mL) was stirred at 90 °C for 16 h. Water was added, and the extraction was performed with EtOAc (50 mL × 3). The combined organic phases were dried over anhydrous MgSO_4_, filtered and concentrated in vacuo. The residue was purified using flash column chromatography (DCM/MeOH = 40:1), followed by flash column chromatography (diethyl ether/EtOAc = 1:20) to afford *tert*-butyl 4-(4-(4-(*p*-tolyloxy)quinolin-7-yl)-1*H*-pyrazol-1-yl)piperidine-1-carboxylate (26.9 mg, 73%). Next, the deprotection of the Boc group was performed with the solution of *tert*-butyl 4-(4-(4-(*p*-tolyloxy)quinolin-7-yl)-1*H*-pyrazol-1-yl)piperidine-1-carboxylate (19.8 mg, 0.0409 mmol) in dichloromethane (0.5 mL) in a 10 mL round bottom flask. After cooling to 0 °C, trifluoroacetic acid (0.3 mL) was added dropwise. The resulting mixture was stirred at room temperature until full conversion was confirmed by TLC on silica gel. The solvent was evaporated in vacuo, and the resulting residue was washed with diethyl ether (1 mL × 3) to afford 7-(1-(piperidin-4-yl)-1*H*-pyrazol-4-yl)-4-(*p*-tolyloxy)quinoline (15.7 mg, 77%).

### Docking simulation

The calculation of binding modes for HS-1308, HS-1336, HS-1338, and HS-1371 on RIP3 was performed with Discovery Studio 4.5 (DS CHARMm-based CDOCKER docking algorithm) using the all-atom model prepared by adding missing atoms to the original X-ray crystal structure. The three-dimensional atomic coordinates required for the docking study were prepared from the X-ray crystal structure of RIP3 (PDB entry: 4M66) in complex with HS compounds under standard conditions (pH 6.5-8.5). The P-loop of the RIP3 ATP binding site is displayed with carbon alpha wires for clarity^31^.

### Enzymatic assays

The inhibitory activities of all compounds toward RIP3 were measured by Reaction Biology Corp (Malvern, PA, USA) by means of radiometric kinase assays ([γ-^32^P]ATP). The enzymatic activity of RIP3 was monitored using 20 μM of myelin basic protein (MBP) dissolved in freshly prepared reaction buffer (20 mM HEPES (pH 7.5), 10 mM MgCl_2_, 1 mM EGTA, 0.02% BRIJ-35, 0.02 mg/mL BSA, 0.1 mM Na_3_VO_4_, 2 mM DTT, 1% DMSO). Each putative RIP3 inhibitor was dissolved in 100% DMSO at specific concentrations and serially diluted with epMotion 5070 in DMSO. Human RIP3 and 20 μM of peptide substrate (MBP) were added to the reaction buffer. After delivering the candidate inhibitor dissolved in DMSO to the kinase reaction mixture using Acoustic technology (Echo550; nanoliter range), the reaction mixture was incubated for 20 min at room temperature. To initiate the enzymatic reaction, ^33^P-ATP with specific activity of 10 μCi/μL was added to the reaction mixture to reach a final ATP concentration of 10 μM. Radioactivity was then monitored using the filter binding method after incubation of the reaction mixture for 2 h at room temperature. At given concentrations of inhibitor, biochemical potency was measured by the percent remaining kinase activity with respect to the vehicle (dimethyl sulfoxide) reaction. Curve fits and IC_50_ values were then obtained using the PRISM program (GraphPad Software). The ATP-competitive inhibitor staurosporine (STSP) was employed as a positive control in this study because of its high biochemical potency against various kinases including RIP3.

### Cell lines and culture conditions

HEK293T, HT-29, H2009, L929, HeLa, and MEF cells were grown in Dulbecco’s Modified Eagle’s Medium (DMEM) supplemented with 10% fetal bovine serum (FBS). RAW264.7 cells were grown in Roswell Park Memorial Institute (RPMI) 1640 media supplemented with 10% FBS. To generate cell lines stably expressing the RIP3 construct, HeLa and H2009 cells were infected with pLX303-hRIP3 lentivirus.

### Antibodies and chemical reagents

Antibodies used in immunoblotting and immunofluorescence: anti-RIP3 (Cell Signaling Technology, 13526s, 1:1000), anti-p-RIP3 (Abcam, ab209384, 1:1000), anti-MLKL (Abcam, ab184718, 1:2000), anti-p-MLKL (Abcam, ab187091, 1:1000), anti-mouse p-MLKL (Abcam, ab196436, 1:5000), anti-RIP1 (BD Biosciences, 610458, 1:1000), anti-p-RIP1 (Cell Signaling Technology, 65746, 1:1000), anti-mouse p-RIP1 (Cell Signaling Technology, 31122, 1:1000), anti-ACTIN (Sigma-Aldrich, A3853, 1:5000), anti-p-ERK (Cell Signaling Technology, 9101s, 1:1000) and anti- IκB-α (Santa Cruz Biotechnology, sc-371, 1:5000). TNF-α and zVAD were purchased from R&D Systems. SMAC mimetic (LCL-161) was purchased from Adooq Bioscience. Dabrafenib and GSK’872 were purchased from Selleckchem. Necrostatin-1, lipopolysaccharide (LPS), and propidium iodide (PI) were purchased from Sigma-Aldrich. Cycloheximide was purchased from Calbiochem. Polyehylenmine was purchased from Polysciences. We purified GST-TRAIL.

### Immunoblot analysis and immunoprecipitation

Cells were rinsed in cold phosphate-buffered saline (PBS) and lysed in M2 buffer containing 20 mM Tris at pH 7, 0.5% NP-40, 250 mM NaCl, 3 mM EDTA, 3 mM EGTA, 2 mM DTT, 0.5 mM PMSF, 20 mM β-glycerol phosphate, 1 mM sodium vanadate, and 1 μg/mL leupeptin. The cell extracts were subjected to western blot analysis. For immunoprecipitation, lysates were mixed and precipitated with antibody and protein A-agarose beads overnight or for 4 h at 4 °C. Bound proteins were removed by boiling in SDS and resolved by SDS-PAGE, and immunoblotting was visualized by enhanced chemiluminescence (ECL, Amersham).

### Cytotoxicity assays

Cell viability was determined using tetrazolium dye colorimetric tests (the MTT assay) read at 570 nm. PI staining was quantified using propidium iodide (Cat. No. 51-66211E, BD Biosciences). Lactate dehydrogenase (LDH) leakage was quantified using the CellTiter-Glo Luminescent Cell Viability Assay kit (Promega, G7570) according to the manufacturer’s instructions. The LDH absorbance was read at 490 nm, and the mean ± STDEV of duplicates is presented.

### Immunofluorescence staining

HT-29 cells were fixed in 4% paraformaldehyde for 10 min. To stain phospho-MLKL, cells were permeabilized with 0.25% Triton X-100 for 10 min. After incubation in a blocking buffer (10% fetal bovine serum in DPBS) for 30 min, the primary antibody to phospho-MLKL was incubated overnight at 4 °C, and FITC-conjugated secondary antibody (goat anti-rabbit IgG, 1:250, dilution, Invitrogen) was incubated for 1 h at room temperature. A mounting medium containing DAPI (VECTASHIELD, Cat No. 94010, Vector Laboratories) was used for counterstaining. Representative images were taken by confocal microscope.

### Lentiviral shRNA experiments

MISSION short-hairpin RNA (shRNA) plasmids targeting hRIP3 mRNA (NM_006871), hMLKL mRNA (NM_152649), and non-targeting control sequences (NM_027088) were obtained from Sigma-Aldrich. Lentiviral plasmids were transfected into 293TN cells (System Biosciences, LV900A-1) using Lipofectamine 2000 (Invitrogen, 11668019). Pseudoviral particles were collected 2 days after the transfection and infected into cells with polybrene (8 μg/mL). Infected cells were puromycin selected two days after infection, and knockdown was confirmed by immunoblotting.

### Reverse transcription-PCR and real-time PCR

Total RNA was extracted using the TRIzol reagent (Life Technologies) according to the manufacturer’s instructions. Total RNA (1 μg) from each sample was converted to cDNA using MMLV reverse transcriptase (MGmed, Seoul, Korea). Equal amounts of cDNA product were used in reverse transcription-PCR conducted using the GoTaq® Green Master Mix (Promega). Real-time PCR amplification was performed using iQ™ SYBR® Green Supermix (Bio-rad). Amplification was performed using the following primers: IL-1β forward (5′-TGCCACCTTTTGACAGTGATG-3′), IL-1β reverse (5′-AAGGTCCACGGGAAAGACAC-3′), IL-6 forward (5′-TCCAGTTGCCTTCTTGGGAC-3′), IL-6 reverse (5′-GTACTCCAGAAGACCAGAGG-3′), TNF-α forward (5′-CGAGTGACAAGCCTGTAGCC-3′), TNF-α reverse (5′-ACAAGGTACAACCCATCGGC-3′), GAPDH forward (5′-GGAGCCAAAAGGGTCATCAT-3′), and GAPDH reverse (5′-GTGATGGCATGGACTGTGGT-3′).

### Statistical analysis

Independent experiments were performed at least in triplicate. Statistical significance was evaluated in paired analyses using the Mann–Whitney *U*-test (nonparametric), depending on the data distribution. Data values are expressed as the mean ± SEM. Statistical significance is defined as *P* < 0.05.

## Results

### HS-1371, a novel inhibitor of RIP3 kinase

**Fig. 1:**
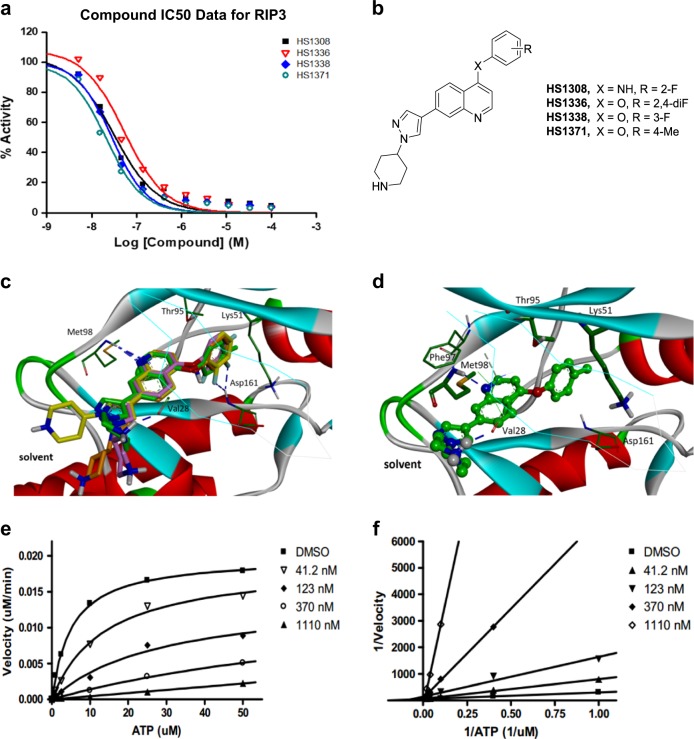
Identification of HS-1371 as a novel RIP3 kinase inhibitor. **a** IC_50_ curves for RIP3 with HS-1308, 1336, 1338, and 1371 treatments. **b** Chemical structures of HS-1308, 1336, 1338, and 1371. **c**, **d**. **c** Comparative view of the predicted binding conformations of HS-1308, 1336, 1338 and 1371 in the ATP-binding site of RIP3 (PDB ID: 4M66). Carbon atoms of HS-1308, 1336, 1338 and 1371 are colored in yellow, pink, brown, and green, respectively. **d** Detailed interaction patterns of HS-1371 in the ATP-binding site of RIP3. Hydrogen-bond interactions are represented with blue dotted lines. **e**, **f**. **e** Michaelis–Menten plot and **f** Lineweaver-Burk Plot for RIP3 with HS-1371

Small molecule RIP3 inhibitors have potential preventive or therapeutic applications in multiple pathological conditions. To search for potent RIP3 inhibitors, we screened our chemical libraries that were focused toward kinases, revealing that quinolone-based compounds combined with a 4-(1H-pyrazol-1-yl)piperidine group at the C7 position exhibited excellent inhibitory activity against RIP3 (HS-1371, IC_50_ = 20.8 nM). The chemical structures and biochemical IC_50_ potencies of the four inhibitors (HS-1308, 1336, 1338, and 1371) are summarized in Fig. 1a, b, respectively. To the best of our knowledge, RIP3 inhibitors bearing a quinoline scaffold have not yet been reported.

Overlaid in Fig. 1c are the docked poses of HS-1308, 1336, 1338, and 1371 around the ATP-binding site of RIP3 (PDB ID: 4M66). All four inhibitors appear to be accommodated in the well-established ATP binding site of RIP3: they involve two hydrogen bonds and van der Waals contacts with residues in the hinge region of RIP3. Figure 1d illustrates the most stable binding mode of HS-1371 derived with Discovery Studio 4.5 software. We note that the nitrogen of the quinoline group receives a hydrogen bond from the backbone amidic nitrogen of Met98. In addition, the piperidine group appeared to form a hydrogen bond with the backbone carbonyl group of Val28. The simulation suggests that the ability of HS-1371 to establish such hydrogen bonds with the backbone groups in the hinge region of RIP3 is necessary for its tight binding in the ATP-binding site of RIP3. Our data also suggest that compound HS-1371 could be further stabilized in the ATP-binding site via a π–π interaction with Phe97 and hydrophobic interactions with Thr95, Asp161 and catalytic Lys51 side chains enclosing the 4-methylphenoxy group in the back pocket.

Despite the overall similarity in RIP3 binding modes, one difference in the interaction features can be observed in the region surrounded by Asp161 (DFG motif) and Glu61 (αC helix). The 4-anilino and 4-phenoxy groups of compounds are oriented toward the space between Thr95 and Lys51 in a direction perpendicular to the quinoline core. Whereas HS-1371 can be further stabilized by favorable hydrophobic interaction with Thr95, Asp161, and catalytic Lys51 residues enclosing the terminal 4-methylphenoxy group of HS-1371, electron-rich F substituents on the aryl group (HS-1308, 1336, and 1338) appear to approach the negatively charged side chains of Asp161 and Glu61 (Supplementary Figure S1).

To understand the interaction modes of these inhibitors within RIP3, the mechanisms of action studies including ATP competition were further performed with HS-1371. A 20 min pre-incubation of the compound and RIP3 enzyme was performed to ensure HS-1371 binding to the enzyme and equilibration. The reactions were monitored every 5–15 min to obtain progress curves with a time course. At each time point, radioisotope signal (^33^P) was converted into M phosphate transferred to substrate and was plotted against time. The slopes (or velocity; M/min) were then plotted against ATP concentrations to generate a Michaelis–Menten plot and a subsequent Lineweaver–Burk plot (double-reciprocal plot) using GraphPad Prism software (GraphPad Software Inc., San Diego, CA). The results were further analyzed with global fit using GraFit software. The apparent *K*_m_ was increased when the concentration of HS-1371 was increased in the Michaelis–Menten plot, and all lines converged on the *Y*-axis in the Lineweaver–Burk plots (Fig. 1e, f), indicating that the mechanism of action was time-independent and ATP-competitive upon binding to RIP3.

### HS-1371 blocks RIP3 kinase activity

**Fig. 2:**
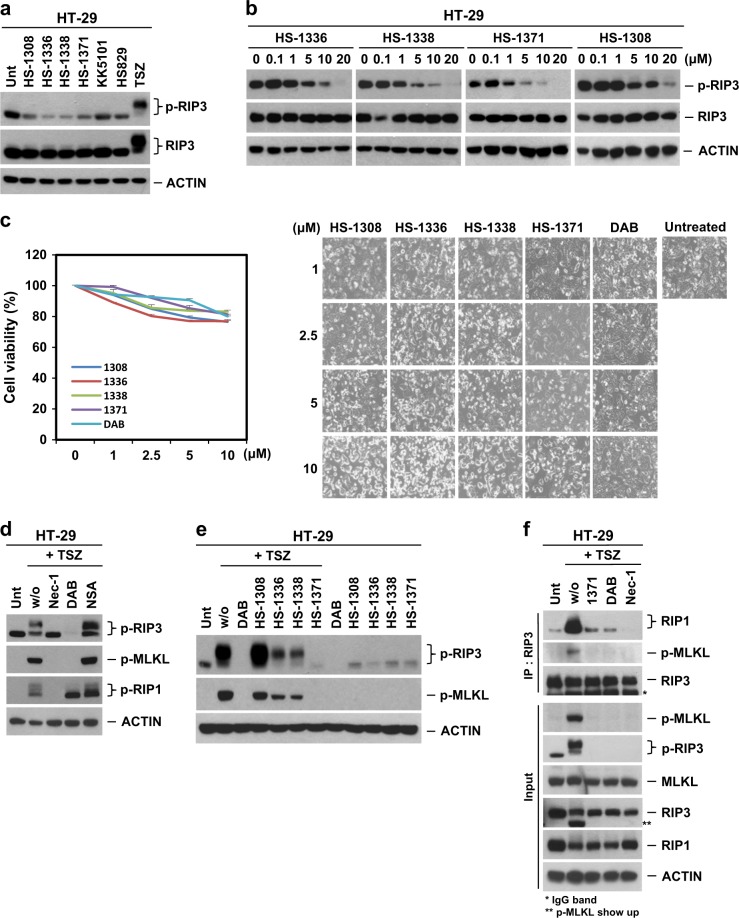
Novel inhibitors can block RIP3 S227 phosphorylation. **a** Western blotting showing various kinase inhibitor effects on RIP3 S227 auto-phosphorylation. HT-29 cells were treated with four tested kinase inhibitors (1308, 1336, 1338, and 1371, 10 μM) for 9 h. As a control, KK5101 (Trk alpha inhibitor) and HS829 (IKK beta inhibitor) were also treated at a concentration of 10 μM for 9 h. Prototypical necroptosis stimuli (TNF + zVAD + either cycloheximide or SMAC mimetic; hereafter referred to as TCZ or TSZ) were applied for 6 h. Cell lysates were analyzed with a S227 specific p-RIP3 antibody. **b** Four tested kinase inhibitors displayed kinase inhibitory effects on basal levels of RIP3 auto-phosphorylation in a dose-dependent manner. HT-29 cells were treated with indicated concentrations for 9 h, and cell lysates were analyzed by immunoblotting. **c** Four tested kinase inhibitors showed small amounts of cytotoxicity in a dose-dependent manner. HT-29 cells were treated with 4 tested kinase inhibitors for 24 h, and cell viability was analyzed by MTT assay or phase-contrast microscopy. The results are presented as means ± SEM. *P *<* 0.05, **P *<* 0.01, ***P *<* 0.001. **d** Various necroptosis inhibitors blocked TNF-induced RIP3 phosphorylation. HT-29 cells were pretreated with necrostatin-1 (Nec-1, 40 μM), dabrafenib (DAB, 10 μM) or necrosulfonamide (NSA, 1 μM) for 2 h and then treated with TSZ for 6 h. Cell lysates were analyzed by immunoblotting. **e** HS-1371 efficiently blocked TNF-induced RIP3 phosphorylation. HT-29 cells were pretreated with four tested kinase inhibitors for 2 h and then treated with TSZ for 6 h. Cell lysates were analyzed by immunoblotting. **f** HT-29 cells were pretreated with Nec-1 (40 μM), DAB (5 μM) or HS-1371 (5 μM) for 2 h and then treated with TSZ for 4 h. Cell lysates were immunoprecipitated with anti-RIP3 antibody. Immunoprecipitates and total lysates were analyzed by Western blotting

Since our data show that all four inhibitors appear to be accommodated in the well-established ATP-binding site and that the mechanism of action is ATP-competitive upon binding to RIP3 in vitro, we would expect that the drug would inhibit RIP3 kinase activity and abolish downstream signaling in an in vivo cell system. RIP3 kinase activity is essential for TNF-induced necroptosis, and human S227 auto-phosphorylation sites of RIP3 are required for the interaction of RIP3 with its substrate MLKL^13,14^, which is indispensable for downstream necroptotic cell death signaling. To test the possible effects of the RIP3 inhibitors on necroptosis, HT-29 cells were treated with the kinase inhibitors, and RIP3 kinase activity was determined by examining its phosphorylation status. Four tested inhibitors (HS-1308, HS-1336, HS-1338, and HS-1371) showed an inhibitory effect on S227 auto-phosphorylation of RIP3 at the basal level, and the other two kinase inhibitors (KK5101 and HS829) had no effect (Fig. 2a). The results were further confirmed in a dose-dependent manner (Fig. 2b), indicating that these four tested compounds can be used as potential preventive agents in RIP3-mediated necroptotic cell death. However, these inhibitors showed a certain degree of cytotoxicity at high concentrations of 10 μM (Fig. 2c).

TNFα Smac mimetic and the caspase inhibitor zVAD (hereafter referred to as TSZ) is a classical combination that is used to induce RIP3-mediated necroptotic cell death^10,32^. We first verified that three established pharmacological inhibitors of necroptosis, necrostatin-1 (Nec-1), necrosulfonamide (NSA), and dabrafenib (DAB) could block TNF-induced necroptosis (Fig. 2d). Of these inhibitors, Nec-1 inhibits RIP1 kinase activity^33,34^, DAB inhibits RIP3 kinase activity^21^, and NSA inhibits MLKL functions downstream of RIP3 phosphorylation^14^. As RIP3 underwent auto-phosphorylation when overexpressed in 293T cells, we further tested an antibody for S227 auto-phosphorylation of RIP3 to determine whether this antibody recognizes a specific band and whether this phosphorylation is inhibited by DAB; this was shown to be the case (Supplementary Figure S2a). Next, we tested whether these four inhibitors would inhibit TNF-induced necroptosis signaling by blocking RIP3 kinase activity. Although all of the tested inhibitors had an inhibitory effect on basal S227 auto-phosphorylation of RIP3, only HS-1371 displayed a complete inhibitory effect on TNF-induced necroptosis signaling, showing no phosphorylation of RIP3 and MLKL, similar to DAB (Fig. 2e). Although these four inhibitors bind to the ATP site of RIP3 *in vitro*, in the cell system, only HS-1371 could potently inhibit RIP3 kinase activity. The effect of HS-1371 on RIP3 kinase activity was compared with another RIP3 inhibitor, GSK’872^16^. As expected, HS-1371 showed a similar level of inhibitory effect on RIP3 kinase activity and cellular cytotoxicity through increased apoptosis when compared with GSK’872 (Supplementary Figure S2b–2d). Inhibition of RIP3 kinase activity by HS-1371 blocked necrosome complex formation, showing disruption of MLKL recruitment (Fig. 2f and Supplementary Figure S2e).

### HS-1371 rescues cells from TNF-induced necroptosis

**Fig. 3:**
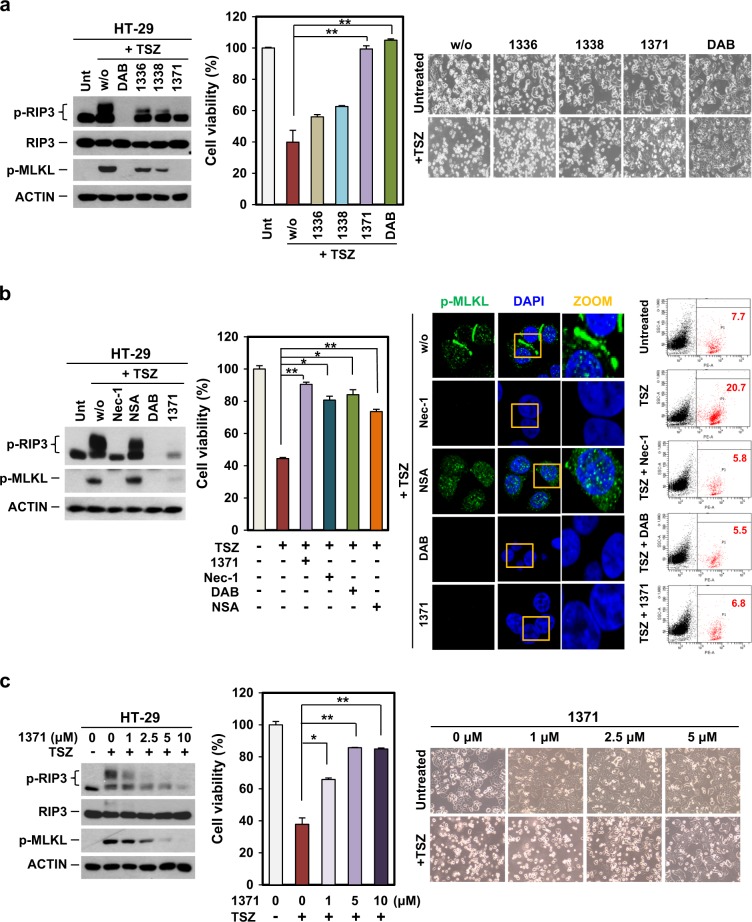
HS-1371 protects cells from TNF-induced necroptosis. **a** TNF-induced necroptosis was completely blocked by HS-1371 treatment. HT-29 cells were pretreated with four tested inhibitors for 2 h and then treated with TSZ (6 h for immunoblotting, 24 h for cell death assay). Cell lysates were analyzed by immunoblotting, and cell viability was analyzed by MTT assay or phase-contrast microscopy. The results are presented as the means ± SEM. *P *<* 0.05, **P *<* 0.01, ***P *<* 0.001. **b** Protection of necroptosis by HS-1371 treatment is similar with dabrafenib, which abolished downstream events. HT-29 cells were pretreated with various necroptosis inhibitors and HS-1371 before TSZ treatment. Cell lysates were analyzed by immunoblotting, and cell viability was analyzed by MTT assay (left panel). Cells were stained with phospho-MLKL antibody and analyzed by confocal fluorescence microscopy (middle panel), and cell death was further analyzed by FACS analysis after PI staining (right panel). **c** HS-1371 protects cells from TNF-induced necroptosis in a dose-dependent manner. HT-29 cells were pretreated with indicated concentrations of HS-1371 and then treated with TSZ (6 h for immunoblotting, 24 h for cell death assay). Cell lysates were analyzed by immunoblotting, and cell viability was analyzed by MTT assay or phase-contrast microscopy. The results are presented as means ± SEM. *P *<* 0.05, **P *<* 0.01, ***P *<* 0.001

We further determined whether these small molecules could rescue cells from TNF-induced cell death. We treated HT-29 cells with these inhibitors followed by TSZ to induce necroptosis. Only HS-1371 rescued TSZ-induced cell death, similar to DAB, and the reduced cytotoxicity was consistent with western blot data showing that HS-1371 effectively blocked RIP3 kinase activity (Fig. 3a). The effect of HS-1371 on TNF-induced cell death was similar to other necroptosis inhibitors, Nec-1, DAB, and NSA (Fig. 3b, left panel). The inhibitory effect on TNF-induced necroptosis was further analyzed by immunofluorescence and FACS. When cells undergo TNF-induced necroptosis, RIP3 phosphorylates MLKL and p-MLKL translocates into the plasma membrane^15,35^. Nec-1, DAB, and HS-1371 prevented RIP1 and RIP3 kinase activity, resulting in no detection of p-MLKL (Fig. 3b, middle panel). NSA functions as an inhibitor of MLKL translocation, but not its phosphorylation, and as expected, TSZ could induce MLKL phosphorylation in the presence of NSA, but the resulting p-MLKL could not translocate into the plasma membrane and stayed in the cytosol. PI-positive cells, which are a marker of membrane permeability during necroptosis, decreased in the presence of HS-1371 in response to TSZ as measured by flow cytometry (Fig. 3b, right panel). Viability in response to TSZ-mediated necroptosis was effectively restored by HS-1371 in a dose-dependent manner (Fig. 3c). Taken together, our results indicate that the novel small molecule HS-1371 acts as a RIP3 inhibitor.

### HS-1371 rescues cells from RIP3-dependent necroptotic cell death but not apoptotic cell death

**Fig. 4:**
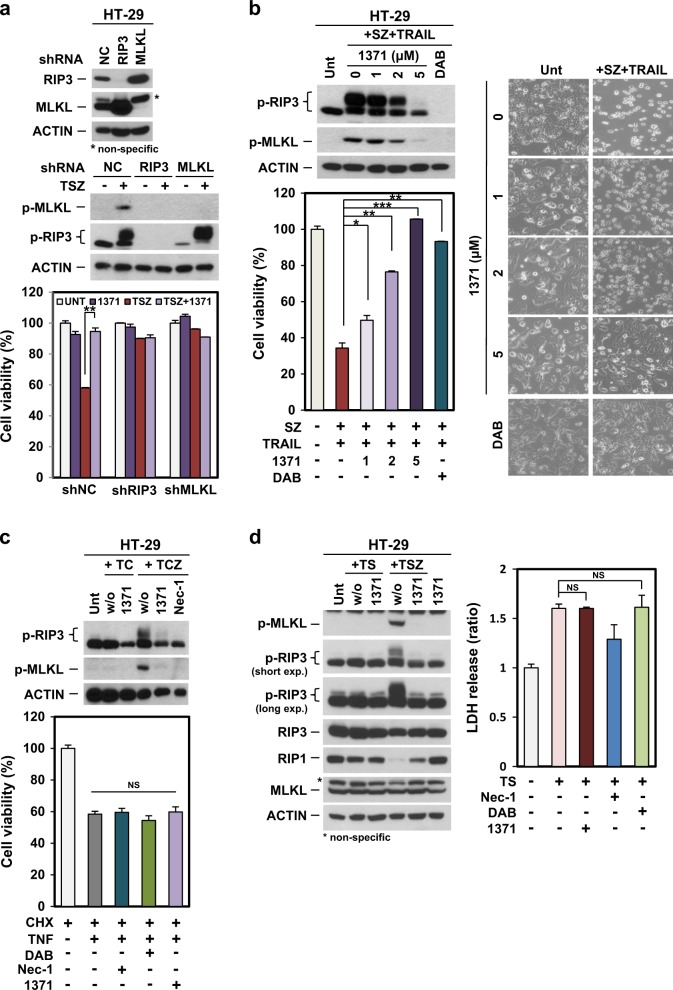
HS-1371 only rescues cells from RIP3-dependent necroptosis. **a** Knockdown of RIP3 had no inhibitory effect on HS-1371 in necroptosis. HT-29 cells expressing RIP3 shRNA, MLKL shRNA, or non-silencing control were analyzed by western blotting (upper panel), and these cells were treated with TSZ (6 h for immunoblotting, 24 h for cell death assay). Cell lysates were analyzed by immunoblotting (middle panel), and cell viability was analyzed by MTT assay (bottom panel). The results are presented as means ± SEM. *P < 0.05, **P < 0.01, ***P < 0.001. **b** HS-1371 protected cells not only from TNF-induced necroptosis but also from TRAIL-induced necroptosis. HT-29 cells were pretreated with indicated concentrations of HS-1371 and then treated with TRAIL + Smac + zVAD (6 h for immunoblotting, 24 h for cell death assay). Cell lysates were analyzed by immunoblotting, and cell viability was analyzed by MTT assay or phase-contrast microscopy. The results are presented as means ± SEM. *P < 0.05, **P < 0.01, ***P < 0.001. **c**, **d** HS-1371 had no effect on TNF-induced apoptosis. HT-29 cells were pretreated with HS-1371 (5 μM) for 2 h and then treated with TC or TS (TNF + either cycloheximide or SMAC mimetic, hereafter referred to as TC or TS, 6 h for immunoblotting, 24 h (TC) or 36 h (TS) for cell death assay). Cell lysates were analyzed by immunoblotting, and cell viability was analyzed by MTT assay (TC) or CellTiter-Glo Luminescent Cell Viability Assay Kit (TS). The results are presented as means ± SEM. *P < 0.05, **P < 0.01, ***P < 0.001

It is possible that the effect of HS-1371 on inhibition of TSZ-mediated necroptosis is an off-target effect and not dependent on regulation of RIP3 kinase activity. To rule out this possibility, RIP3-expressing HT-29 cells were infected with either an shRIP3 or shMLKL lentiviral plasmid to knockdown RIP3 and MLKL, respectively. Knockdown efficiency was analyzed by western blotting (Fig. 4a, upper panel), and these cells were treated with TSZ to examine necroptosis signaling (Fig. 4a, middle panel). HS-1371 did not obviously affect cell viability in either RIP3 or MLKL-deficient cells (Fig. 4a, bottom panel), suggesting that the effect of HS-1371 in preventing TNF-induced necroptosis is dependent on RIP3 kinase activity.

In addition to TNF, various stimuli have also been shown to induce necroptosis. Next, we tested whether HS-1371 could block TRAIL-induced necroptosis. As shown in Fig. 4b, HT-29 cells were treated with TRAIL plus Smac mimetic and zVAD to induce TRAIL-mediated necroptosis; HS-1371 decreased TRAIL-induced S227 phosphorylation of RIP3 and RIP3-mediated phosphorylation of MLKL in a dose-dependent manner. Reduced phosphorylation was consistent with a reduction in cytotoxicity. These data indicate that RIP3-mediated necroptosis could be inhibited by HS-1371. However, HS-1371 did not inhibit apoptosis induced by TNF plus CHX (Fig. 4c) or apoptosis induced by TNF plus Smac mimetic (Fig. 4d), which does not require RIP3 kinase activity, suggesting that this novel inhibitor has a specific inhibitory effect on RIP3-mediated necroptosis.

### Inhibition of RIP3 kinase activity by HS-1371 in various cells

**Fig. 5:**
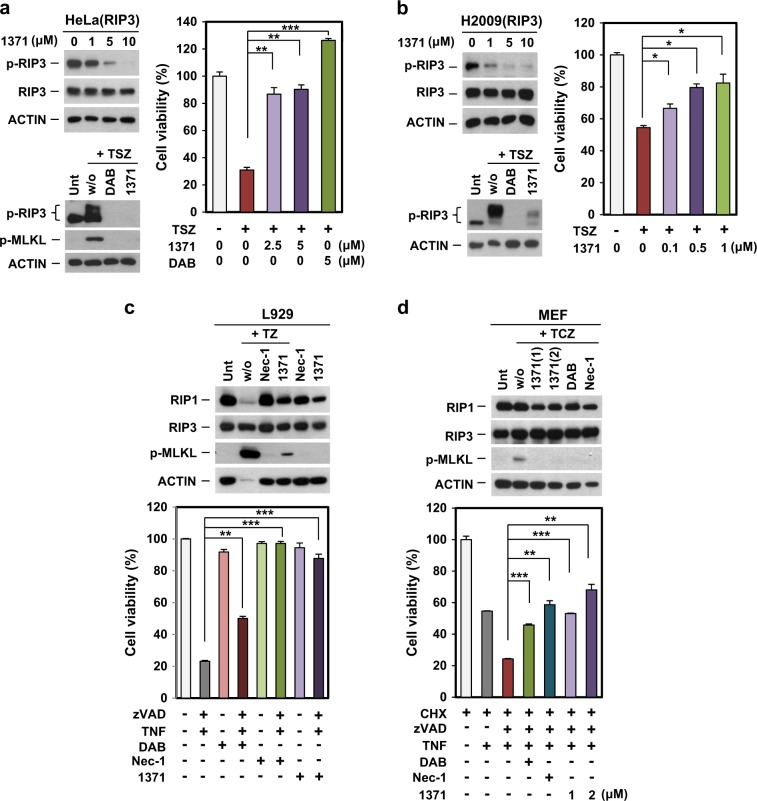
HS-1371 had an inhibitory effect on RIP3 kinase activity in various cell lines. **a**, **b** HeLa (cervical cancer, **a**) and H2009 (lung cancer, **b**) cells ectopically expressing RIP3 were treated with indicated concentrations of HS-1371 for 6 h, and cell lysates were analyzed by immunoblotting (left upper panel). Cells were pretreated with DAB or HS-1371 for 2 h and then treated with TSZ (6 h for immunoblotting, 24 h for cell death assay). Cell lysates were analyzed by immunoblotting (left bottom panel), and cell viability was analyzed by MTT assay (right panel). The results are presented as means ± SEM. *P *<* 0.05, **P *<* 0.01, ***P *<* 0.001. **c**, **d** HS-1371 had the same effect on mouse cells. L929 (mouse sarcoma, **c**) and MEF (mouse embryonic fibroblast, **d**) cells were pretreated with inhibitors and then necroptosis was induced by TNF. Cell lysates were analyzed by immunoblotting (upper panel), and cell viability was analyzed by MTT assay (bottom panel). The results are presented as means ± SEM. *P < 0.05, **P < 0.01, ***P < 0.001

Previously, we reported that most cancer cells do not express RIP3 due to methylation-dependent silencing^18,19^; therefore, we tested whether ectopically expressed RIP3 was also affected by HS-1371 in RIP3-mediated necroptosis. In HeLa cells that lack endogenous RIP3 expression, the ectopic expression of RIP3 resulted in basal activation of RIP3 phosphorylation; this activity was decreased by HS-1371 in a dose-dependent manner (Fig. 5a, left upper panel). RIP3 phosphorylation was markedly increased by TSZ treatment, but pretreatment of HS-1371 effectively blocked RIP3 phosphorylation and MLKL phosphorylation (Fig. 5a, left bottom panel). Consistent with effects on phosphorylation status, cell viability was gradually increased in a dose-dependent manner (Fig. 5a, right panel). HS-1371 also prevented TSZ-induced necroptosis in H2009 cells, which have very low expression levels of endogenous RIP3, with ectopic expression of RIP3 (Fig. 5b). The data further support the evidence that HS-1371 acts as a RIP3 inhibitor and will lead to potential preventive or therapeutic uses in multiple pathological states.

Importantly, HS-1371 also prevented TNF plus zVAD-induced necroptosis in the mouse fibrosarcoma cell line, L929 (Fig. 5c), suggesting that the inhibitor blocks activity of both mouse and human RIP3. Additionally, HS-1371 also effectively prevented MLKL phosphorylation and cell death in mouse embryonic fibroblast (MEF) cells treated with TNF plus cycloheximide (CHX; protein synthesis inhibitor) and zVAD (hereafter referred to as TCZ) (Fig. 5d).

### Post-treatment of HS-1371 also has an inhibitory effect on RIP3-mediated necroptosis

**Fig. 6:**
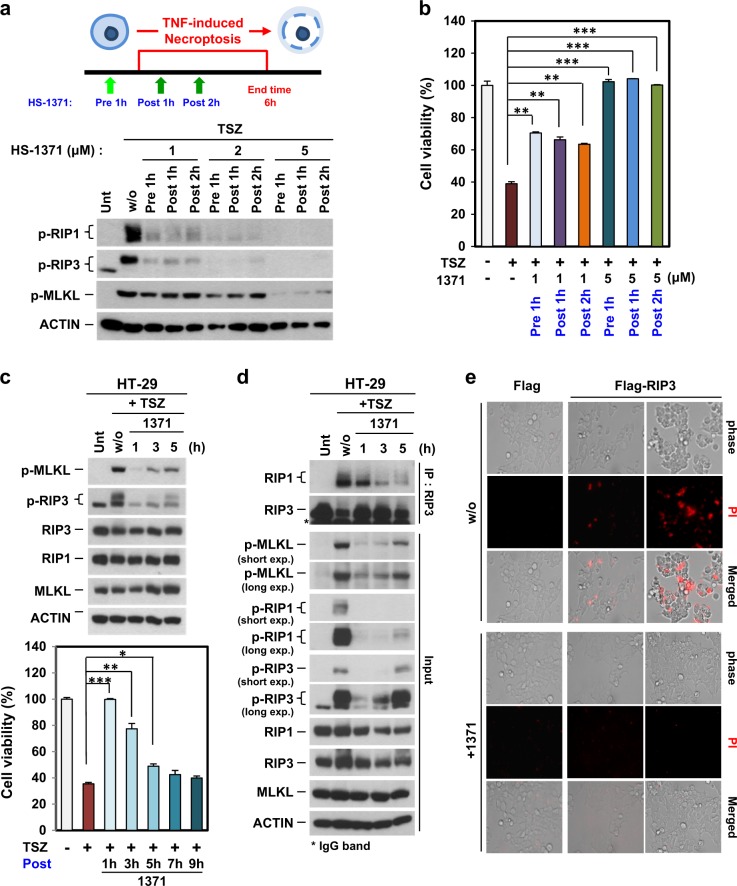
Possibility of HS-1371 as a drug for necroptosis-related diseases. **a**, **b** HT-29 cells were pre- or post-treated with HS-1371 for indicated time points, and RIP3 phosphorylation (**a**) and cell death (**b**) were measured by western blotting or MTT assay. Data are presented as means ± SEM. *P < 0.05, **P < 0.01, ***P *<* 0.001. **c** HT-29 cells were treated with TSZ and then post-treated with HS-1371 for indicated time points, and then cell lysates were analyzed by immunoblotting (upper panel). Cell viability was analyzed by MTT assay. Data are presented as means ± SEM. *P < 0.05, **P < 0.01, ***P < 0.001. **d** Cells were treated with the same conditions as (**c**), and cell lysates were immunoprecipitated with anti-RIP3 antibody. Immunoprecipitates and total lysates were analyzed by Western blotting. **e** Upregulated RIP3 expression-induced cell death was inhibited by treatment with HS-1371. 293T cells were transfected with Flag vector or Flag-RIP3, and 20 h after transfection, cells were stained with PI. PI-positive cells were analyzed by phase-contrast fluorescence microscopy

We tested the inhibitory effect of HS-1371 before and after RIP3 activation. HT-29 cells were pretreated with HS-1371 for 1 h, and then TNF-mediated necroptosis was induced. Alternatively, TNF-mediated necroptosis was induced and then HS-1371 was applied 1 or 2 h later. As shown in Fig. 6a, b, low concentrations of HS-1371 showed a partial inhibitory effect on RIP3 phosphorylation in both pre-treatment and post-treatment, but 5 μM HS-1371 completely inhibited RIP3 phosphorylation and TNF-induced cell death. Since HS-1371 was able to suppress necroptosis after this process was initiated (Fig. 6a, b), we further analyzed the time point at which HS-1371 could no longer prevent cell death. Until after approximately 5 h of treatment, MLKL phosphorylation was partially blocked, and cellular cytotoxicity was significantly reduced (Fig. 6c); this phenomenon was corresponded with necrosome complex formation (Fig. 6d), indicating that HS-1371 may serve as a potential therapeutic candidate for necroptosis-related diseases.

It has been reported that high or upregulated RIP3 expression can lead to spontaneous auto-phosphorylation, then potentiating MLKL mediated necroptotic cell death in keratinocytes from TEN patients^26^. We also tested whether HS-1371 blocks upregulated RIP3 expression-mediated cell death. As shown in Fig. 6e, RIP3 ectopic expression led to cell death detected by PI-positive staining, and this was completely blocked by HS-1371 treatment, suggesting that HS-1371 could effectively inhibit necroptosis induced by RIP3 overexpression. Another possible application model for this drug would be sepsis. Several reports suggest the clinical relevance of RIP3 kinase inhibition in sepsis^36^, which is a high mortality pathological condition that is classified as uncontrollable. TNF, LPS, and other microbial compounds are involved in the pathogenesis of sepsis, leading to tissue damage^37^. Management of the overwhelming inflammatory response is an important issue for the treatment of sepsis, so we tested the anti-inflammatory activity of HS-1371 under LPS-induced septic shock conditions. Activation of the TNFR-1 and TLR (Toll-like receptor)-4 signaling pathway plays an important role in regulating the secretion of pro-inflammatory cytokines through the NF-κB pathway. Because RIP3 kinase activity is not required for the NF-κB pathway, HS-1371 did not affect TNF-induced NF-κB signaling in various cells (Supplementary Figure S3). Similarly, LPS-induced induction of the NF-κB pathway was not altered (Figure S4a and data not shown); however, systematic inflammation contributed by RIP3-mediated necroptosis was inhibited by HS-1371, which was associated with reduced IL-1β, IL-6, and TNF-α expression in RAW264.7 macrophage cells (Supplementary Figure S4b–4e). These results suggest that by using HS-1371 to specifically target necroptosis, it may be possible to therapeutically target sepsis.

## Discussion

The study of cell death mechanisms is important to understand the ultimate outcome of a pathological process, whether cell death is induced by pathogen infection, cancer therapeutics, or any other stressor^1,4,5,8^. Indeed, identification of a cell death mechanism is also important if one seeks to intervene therapeutically to inhibit or enhance a given cell death process. In cancer cells, necroptosis is an alternative cell death pathway; for this process, RIP3 is required, and its kinase activity is necessary to form a stable necrosome complex to propagate necroptosis^38^. Conversely, in normal cells, high RIP3 expression can lead to spontaneous auto-phosphorylation and inopportune necroptosis^39^. Because inhibition of RIP3 kinase activity shows beneficial effects in certain pathological settings but cancer therapies may require RIP3 activity to induce necroptosis, regulation of kinase activity should be considered depending on the drugs used as therapeutic targets.

In this study, we systematically tested four kinase inhibitors and found that they have similar structures; their binding activity to RIP3 is also similar in an in vitro assay system, but HS-1371 showed a much stronger inhibitory effect on RIP3 kinase activity in an in vivo cellular system. HS-1371 is a kinase inhibitor that has a potential use for kinase-dependent cell death, but in our study, we identified this compound as a new potent RIP3 inhibitor, thereby highlighting the risk of misinterpretation when developing drugs as selective kinase inhibitors in cancer therapy^40^. Dabrafenib in combination with MEK inhibitors has already yielded impressive results, but dabrafenib also blocks necroptosis by interference with RIP3 kinase activity^41^, suggesting that the selection of kinase inhibitors to target cancer cell death must consider how cell death machinery is involved and the RIP3 expression pattern in cancer cells. In contrast to dabrafenib, another BRAF inhibitor vemurafenib, which also suppresses proliferation of BRAF-mutated melanoma cells, does not suppress RIP3 activity, indicating that these kinase inhibitors act similarly, but their effect on cancer cells may differ depending on cellular context.

RIP3 is widely involved in physiological and pathological processes, and new RIP3 inhibitors can be used as probes to explore the roles of RIP3 enzymatic activity in addition to their potential applications in RIP3 hyperactivation-associated pathological settings such as inflammatory bowel disease, chronic obstructive pulmonary disease, multiple sclerosis, and toxic epidermal necrolysis.

## Electronic supplementary material

Supplentary Data 1-4
