# Supplementary material for: HS-1371, a novel kinase inhibitor of RIP3-mediated necroptosis
Source: Exp Mol Med. 2018 Sep 20;50(9):125. doi: 10.1038/s12276-018-0152-8 (PMC6148246; doi:10.1038/s12276-018-0152-8)
Supplement: Supplementary file 1 — Supplentary Data 1-4 [file 12276_2018_152_MOESM1_ESM.pdf]

Supplementary Figure 1.

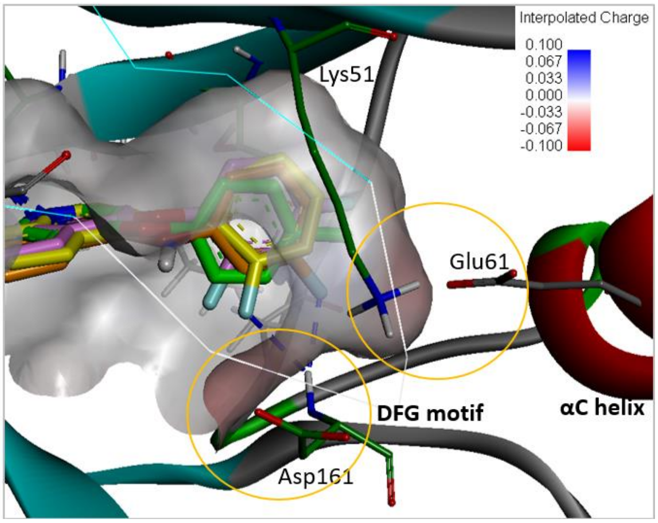

Supplementary Figure 2.

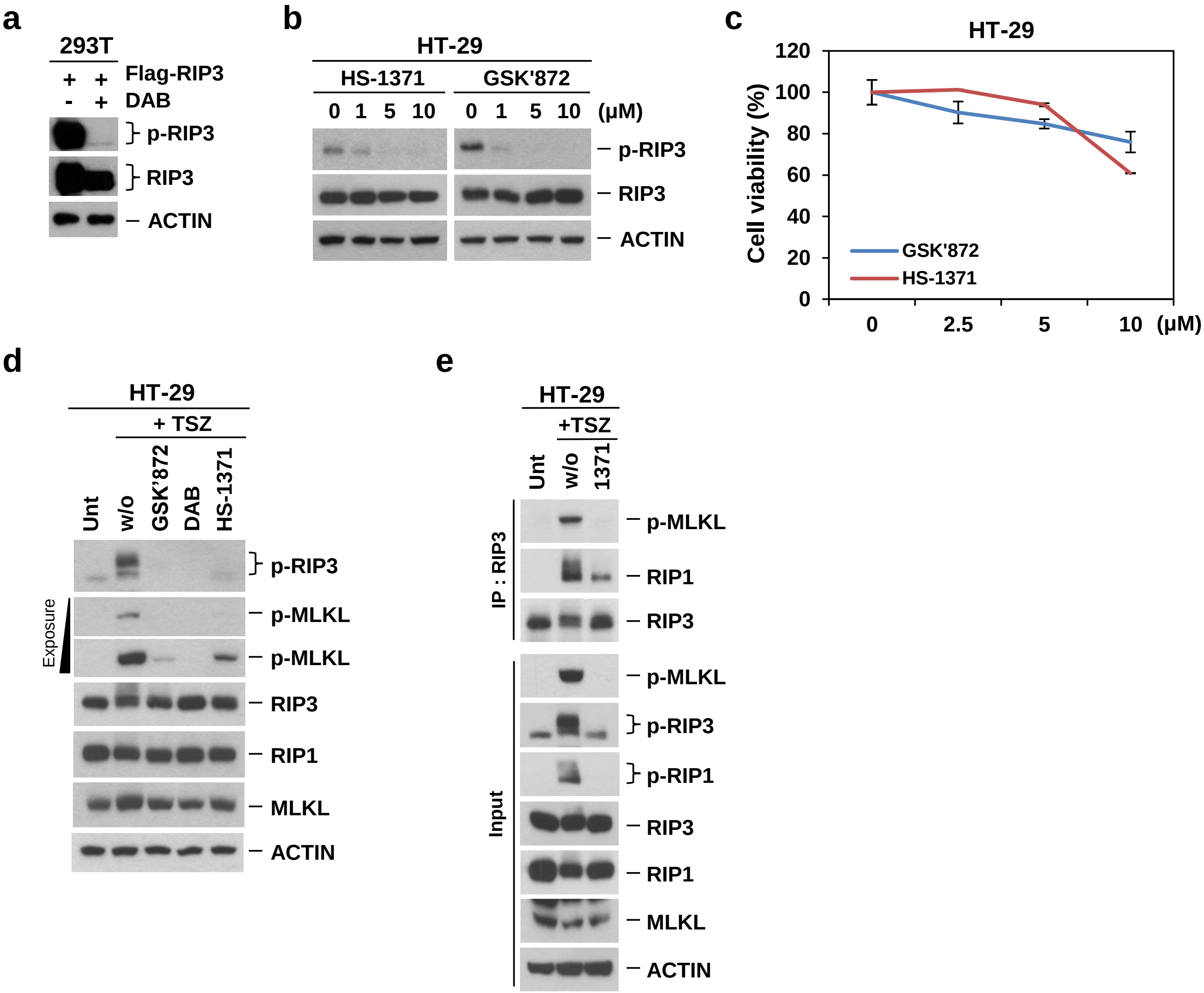

Supplementary Figure 3.

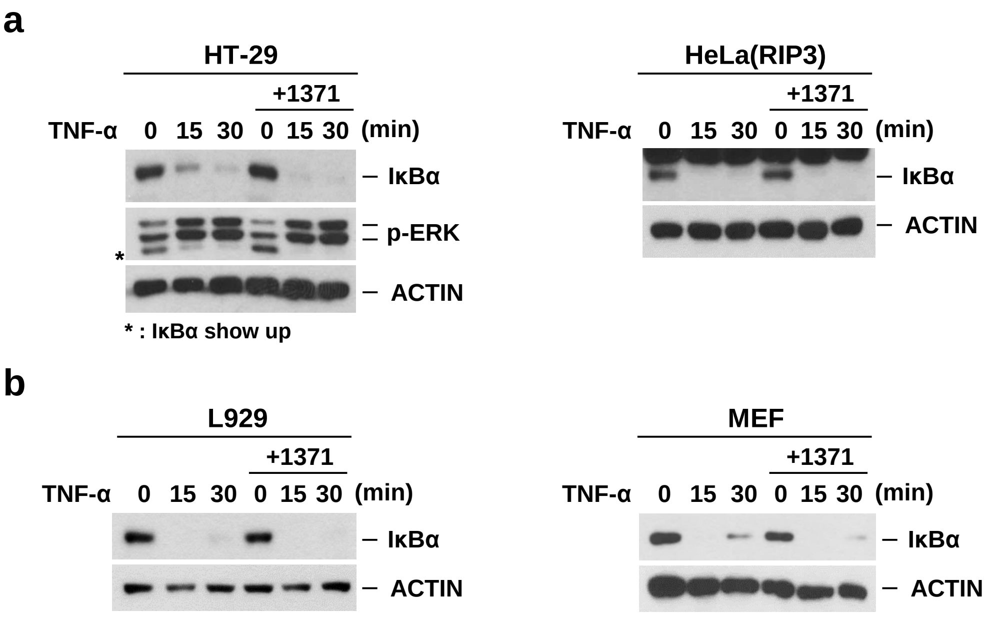

Supplementary Figure 4.

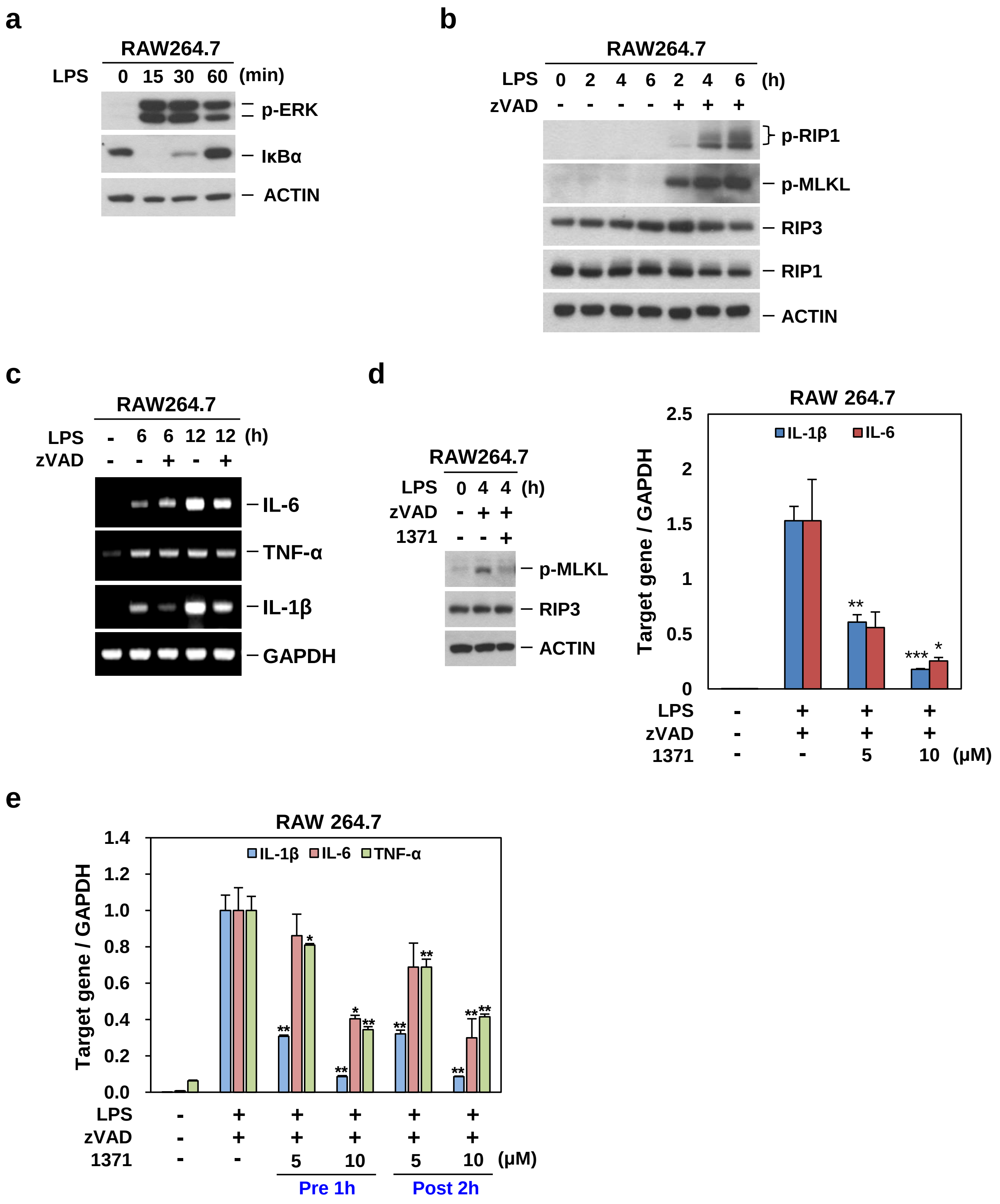

SUPPLEMENTARY FIGURE LEGENDS

Supplementary Figure 1. Identification of HS-1371 as a novel RIP3 kinase inhibitor.

Detailed interaction patterns in the region surrounded by DFG motif and αC helix of RIP3. Hydrogen bonds are indicated with dotted lines.

Supplementary Figure 2. The effect of HS-1371 was compared with GSK'872.

(a) Overexpression of RIP3 could induce S227 phosphorylation without stimuli. HEK293T cells were transiently transfected with Flag-hRIP3, and cells were treated with or without DAB for incubation periods. After cells were harvested, cell lysates were analyzed by immunoblotting.

(b) Kinase inhibitory effect of HS-1371 was compared with GSK'872 which is known RIP3 kinase inhibitor. HT-29 cells were treated with HS-1371 or GSK'872 indicated concentrations for 9 h, and cell lysates were analyzed by immunoblotting.

(c) Cytotoxicity of HS-1371 was compared with GSK'872. HT-29 cells were treated with 2 kinase inhibitors for 24 h and cell viability was analyzed by MTT assay.

(d) Inhibition of MLKL phosphorylation by HS-1371 was compared with other two RIP3 kinases inhibitors, DAB (5 μM) and GSK'872 (5 μM). HT-29 cells were pretreated with 3 kinase inhibitors for 2 h and then treated with TSZ for 6 h. Cell lysates were analyzed by immunoblotting.

(e) HS-1371 inhibits necrosome complex formation. HT-29 cells were pretreated with HS-1371 (5 μM) for 2 h and then treated with TSZ for 4 h. Cell lysates were immunoprecipitated with anti-RIP3 antibody.

Supplementary Figure 3. HS-1371 had no effect on TNF-induced NF-κB signaling.

(a & b) HS-1371 had no effect on TNF-induced NF-κB signaling. HT-29, RIP3 expressing

HeLa, L929, and MEF cells were pretreated with HS-1371 (5 μM) for 2 h, and treated with TNF (30 ng/mL) for indicated time points. Cell lysates were analyzed by immunoblotting.

Supplementary Figure 4. Necroptosis-mediated inflammatory response was decreased by HS-1371.

(a) RAW264.7 cells were treated with LPS (1 μg/mL) for indicated time points and cell lysates were analyzed by immunoblotting.

(b) RAW264.7 cells were pretreated with zVAD (20 μM) for 1 h and then treated with LPS (1 μg/mL) for indicated time points. Cell lysates were analyzed by immunoblotting.

(c) IL-6, TNF-α, and IL-1β expression levels were determined by RT-PCR analysis. RAW264.7 cells were treated with LPS alone or LPS plus zVAD. GAPDH is shown as an internal control.

(d) RAW264.7 cells were pretreated with zVAD (20 μM) and HS-1371 (5 μM) for 1 h and then treated with LPS (1 μg/mL) for 4 h. Cell lysates were analyzed by immunoblotting (left panel). IL-1β and IL-6 expression levels were determined by real-time PCR analysis. RAW264.7 cells were pretreated with zVAD and HS-1371 indicated concentrations for 1 h and then treated with LPS for 12 h (right panel).

(e) IL-1β, IL-6 and TNF-α expression levels were determined by real-time PCR analysis. RAW264.7 cells were treated with HS-1371 (pretreatment for 1 h or post-treatment for 2 h). zVAD were pretreated 1 h before LPS treatment (12 h).
